# Venovenous Extracorporeal Membrane Oxygenation After Cardiac Arrest Due to Refractory Anaphylaxis From Nut Ingestion

**DOI:** 10.1097/CCE.0000000000001403

**Published:** 2026-04-15

**Authors:** Catherine Yip, Katherine Hickey, Joanne Hojsak, Ariel Brandwein, Shunpei Okochi

**Affiliations:** 1 Department of Surgery, Icahn School of Medicine at Mount Sinai, New York, NY.; 2 Department of Pediatric Critical Care, Mount Sinai Kravis Children’s Hospital, New York, NY.

**Keywords:** anaphylaxis, pediatric, venovenous extracorporeal membrane oxygenation

## Abstract

**BACKGROUND:**

Severe anaphylaxis can cause vasoplegia, decreased cardiac output, bronchoconstriction, and acute pulmonary edema, leading to cardiopulmonary arrest. In evaluating these patients for extracorporeal membrane oxygenation (ECMO), it is important to determine the principal factor underlying the patient’s failure of medical management. In this case, we describe the successful use of venovenous ECMO in a pediatric patient with anaphylactic shock and cardiac arrest.

**CASE SUMMARY:**

A 15-year-old male presented after anaphylaxis leading to a 45-minute cardiac arrest. Although return of spontaneous circulation (ROSC) was achieved while preparing for extracorporeal cardiopulmonary resuscitation, he had profound hypoxic respiratory failure from pulmonary edema with an oxygenation index of 45, and he required vasoactive support with epinephrine and norepinephrine. With signs of adequate perfusion and an improving pulse pressure gradient from 10 to 30, a rapid decision was made to change the cannulation strategy from venoarterial to venovenous ECMO. He was decannulated 3 days later with resolution of his acute respiratory failure, extubated 5 days later, and discharged home neurologically intact 12 days later.

**CONCLUSIONS:**

The periarrest decision to modify the cannulation strategy demonstrates that, even after prolonged cardiac arrest and ROSC, venovenous ECMO can be a safe, potential bridge to the resolution of pulmonary edema, with careful consideration of individual patient characteristics.

**KEY POINTS**

**Question**: The goal of this case report is to describe an unconventional approach to treating a pediatric patient after cardiac arrest resulting from anaphylaxis.

**Findings**: In this case, we present the decision-making process underlying a rapid change in cannulation strategy from venoarterial to venovenous extracorporeal membrane oxygenation (ECMO) in a 15-year-old male who suffered cardiac arrest stemming from refractory anaphylaxis with subsequent severe pulmonary edema. The patient was cannulated for 3 days, then was ultimately able to be discharged home neurologically intact after 12 days.

**Meaning**: Venovenous ECMO is a viable option after cardiac arrest in cases of severe refractory anaphylaxis with careful consideration of each patient’s physiologic status.

Anaphylaxis is a life-threatening acute reaction involving two or more organ systems following allergen exposure (1, 2). It results from immunoglobulin E-mediated mast cell and basophil activation after allergen exposure, triggering the release of histamines, tryptases, and other bioactive molecules that cause severe vasodilation and ultimately shock (3). Approximately 40% of children experience severe reactions, characterized by airway edema, dyspnea, and hypotension (4, 5).

Epinephrine is the first-line treatment (2), yet symptoms in severe refractory cases can persist after administration. Pulmonary edema, caused by increased pulmonary vascular permeability, is one such symptom exacerbating hypoxia. Consequently, anaphylaxis can require rapid escalation to mechanical ventilation and even extracorporeal support (6). Extracorporeal membrane oxygenation (ECMO) has been described in refractory anaphylaxis. However, venoarterial ECMO is the primarily reported modality (7–18) after cardiac arrest, with only a few reports of venovenous ECMO in this setting (18, 19). Here, we discuss a 15-year-old patient successfully managed with venovenous ECMO after cardiac arrest stemming from refractory anaphylactic shock.

## CASE

A 15-year-old male with known anaphylaxis to multiple foods presented to an outside hospital emergency department after inadvertent nut ingestion. Despite receiving diphenhydramine and intramuscular epinephrine before presentation, diffuse urticaria and labored breathing persisted upon arrival. He received two additional doses of intramuscular epinephrine (0.3 mg each), methylprednisolone (125 mg), IV diphenhydramine (50 mg), and famotidine (20 mg) with only transient improvement, followed by worsening dyspnea and emesis. He was given another dose of intramuscular epinephrine (0.3 mg), started on terbutaline (0.2 µg/kg/min) and epinephrine (0.2 µg/kg/min) infusions, and placed on supplemental oxygen. Due to continued decompensation and concern for impending airway collapse, he was intubated and urgent transfer to our institution was initiated.

Following intubation, the patient was persistently hypoxic with poor pulmonary compliance requiring manual bag-mask ventilation to overcome high airway pressures and achieve adequate tidal volumes. Chest radiographs demonstrated severe bilateral pulmonary edema. Upon arrival of the transport team, he required a Fio_2_ of 100% and positive end-expiratory pressure (PEEP) of 20, with an oxygenation index (OI) of 31.8. Epinephrine infusion was at 0.25 µg/kg/min and norepinephrine at 0.15 µg/kg/min. Arterial blood gas demonstrated a mixed respiratory and metabolic acidosis with pH 6.89, Paco_2_ 87, and lactate of 5 mmol/L. During transport, the patient suffered cardiac arrest and cardiopulmonary resuscitation (CPR) was initiated. The ECMO team was notified for extracorporeal CPR (ECPR), and he was directly brought to the PICU for resuscitation.

**Figure 1.**
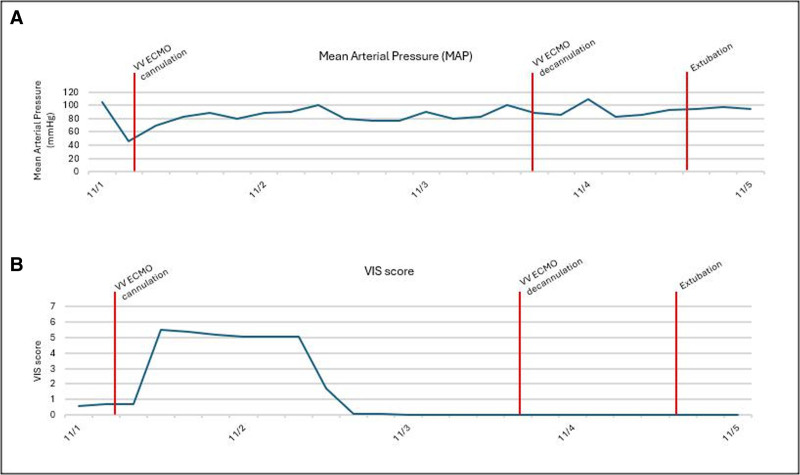
Trend of cardiovascular characteristics before and after venovenous (VV) extracorporeal membrane oxygenation (ECMO) cannulation. Blood pressure in the form of mean arterial pressure (MAP, **A**) and Vasoactive-Inotropic Score (VIS, **B**) improved after VV ECMO cannulation. With stable pre-cannulation vasopressor support with norepinephrine, epinephrine, and vasopressin, ECMO cannulation allowed for weaning of all vasopressors within 8 hr of cannulation.

On arrival, he was in pulseless electrical activity arrest and the ECMO team immediately began preparing for ECPR cannulation. Return of spontaneous circulation (ROSC) was briefly achieved; however, he developed a wide complex tachycardia leading to recurrent cardiac arrest. ROSC was again achieved with CPR and defibrillation as the ECMO team prepared to cannulate. Cumulative arrest time was 45 minutes. Immediate post-arrest chest radiograph demonstrated worsening pulmonary edema and echocardiogram demonstrated mildly decreased right ventricular function and severely decreased left ventricular (LV) function, with an estimated LV ejection fraction (LVEF) 33%. At this point, the patient demonstrated a mixed metabolic and respiratory acidosis (lactate 13.5 mmol/L) with epinephrine at 0.25 µg/kg/min and norepinephrine at 0.15 µg/kg/min. His OI was 45, and he required maximum ventilatory settings (Fio_2_ 100%, PEEP 16). On examination, he had warm, well-perfused extremities, capillary refill under 3 seconds, and a stable vasopressor requirement (**Fig. 1**). He also had improving pulse pressures from 10 to 30, reactive pupils bilaterally, and intact brainstem reflexes. With these considerations, the cannulation approach was changed to percutaneous dual-lumen venovenous ECMO with preparations to add an arterial cannula afterward if necessary.

Following venovenous ECMO cannulation, oxygenation and ventilation rapidly improved, but the metabolic acidosis persisted. Three hours after cannulation, ventilator settings were Fio_2_ 50% and PEEP 15; however, pH was 7.09, and lactate was 13 mmol/L. The patient had multisystem organ dysfunction including shock liver and oliguric acute kidney injury, and continuous renal replacement therapy was initiated. Over the next 24 hours, both the lactic acidosis and pulmonary edema began improving (**Supplemental Fig**., https://links.lww.com/CCX/B619), allowing for further ventilator weaning. Echocardiogram on ECMO day 2 demonstrated improving biventricular function. On ECMO day 3, he was weaned off all pressors and the ECMO sweep gas was turned off. After tolerating a trial off ECMO, he was successfully decannulated. Echocardiograms demonstrated further improvement in cardiac function with LVEF 42% after decannulation and LVEF 50% on post-decannulation day 4. Ventilatory support was further weaned, and he was extubated on hospital day 5. On transfer to the regular floor, he was neurologically intact with minimal recollection of the event. His transaminase levels continued to normalize and urine output slowly improved. He was discharged home on hospital day 15 and ultimately had complete hepatic and renal recovery at subsequent follow-up.

## DISCUSSION

In this case, the patient suffered complete cardiorespiratory collapse due to anaphylaxis from inadvertent ingestion of nuts. During ongoing chest compressions, the ECMO team prepared for ECPR cannulation. Despite obtaining ROSC, he had profound respiratory compromise due to acute pulmonary edema and ventilator pressures impeding venous return to the right heart. With signs of improving perfusion after ROSC, a rapid decision was made to change from a venoarterial to a percutaneous venovenous ECMO approach. While, in hindsight, this case is reminiscent of pediatric patients with severe pulmonary edema managed on venovenous ECMO, its novelty stems from the rapid consideration of the patient’s physiology and risks and benefits of both modalities before changing cannulation strategy.

Multiple factors led to the decision for venovenous ECMO. The OI of 45 highlighted the need for respiratory extracorporeal support, and venovenous ECMO improves both oxygenation and ventilation (20). The patient demonstrated improving diastolic blood pressure and pulse pressure gradient after ROSC, underscoring his cardiac recovery. His vasopressor requirement remained stable with evidence of adequate perfusion, suggesting that myocardial function would continue recovering with improvements in oxygenation, preload, and acidemia. Indeed, following ECMO cannulation, his mean airway pressures, acidosis, and ventilatory and pressor requirements noticeably improved.

Second, the risk of neurologic injury was a chief concern. Although most studies demonstrating increased neurologic complications in venoarterial vs. venovenous ECMO are in the adult population (21), similar patterns exist in pediatric and neonatal populations (22). Given our patient’s age, size, and increased stroke risk with carotid cannulation at his age (23), our strategy for venoarterial ECMO would have been femoral cannulation. However, North-South syndrome, characterized by differential oxygenation to the upper and lower body in the setting of persistently poor pulmonary function, was a consideration with femoral cannulation. Recovering native cardiac function competes against the peripheral inflow of well-oxygenated blood from the venoarterial ECMO circuit and circulates poorly oxygenated blood to the brain and upper body, thus increasing the risk of additional secondary ischemic insult (24). As our patient was 15 years old and demonstrating cardiac recovery, this phenomenon augmented the risk of neurologic complications associated with venoarterial ECMO.

A third consideration was the greater morbidity of femoral artery cannulation compared with percutaneous venous cannulation. Limited mobility, groin hematoma, and acute limb ischemia are among the known complications associated with femoral artery cannulation (25). Although percutaneous venous cannulation is not risk-free, the overall complication rate is lower than femoral artery cutdown (26).

Previous literature describes venovenous ECMO in cases of refractory anaphylaxis; however, pediatric cases involving cardiac arrest are limited. Chan-Dominy et al (18) describe a 12-year-old patient initially cannulated onto venovenous ECMO after cardiac arrest due to anaphylaxis from peanut exposure. However, the patient was precipitously converted to venoarterial ECMO after developing ventricular fibrillation refractory to defibrillation and amiodarone. Frederick et al (19) highlight a 16-year-old female cannulated onto venovenous ECMO after anaphylaxis from peanuts, leading to a 5-minute cardiac arrest. Although consideration of venoarterial ECMO was not discussed, the patient was successfully decannulated after 36 hours with full cardiac recovery and without neurologic impairment, highlighting that venovenous ECMO is possible in the appropriate setting. Venovenous ECMO has also been used in adults with acute refractory anaphylaxis (27–29); however, these cases did not involve cardiac arrest.

While pediatric acute respiratory distress syndrome is a known indication for venovenous ECMO, preceding cardiac arrest complicates the determination of cannulation strategy. ELSO guidelines support trialing venovenous cannulation in pediatric respiratory ECMO if the patient requires minimal to modest inotropic or vasopressor support at the time of cannulation (30). However, these guidelines do not address the post-arrest setting. In a retrospective study of 21 adults cannulated onto venovenous ECMO after cardiac arrest, Bhardwaj et al (31) demonstrated a 57% survival rate, comparable to survival rates of 57% and 64% for adult and pediatric respiratory ECMO, respectively (30). This suggests venovenous ECMO may be appropriate in select patients after cardiac arrest stemming from respiratory causes, so long as they demonstrate adequate cardiac recovery, sparing them from the increased complications associated with venoarterial ECMO. However, there are no such comparative studies in the pediatric population, as venoarterial ECMO is the overwhelmingly used modality in the context of cardiopulmonary arrest, defined as ECPR (32). As such, further studies are needed to examine the pediatric post-arrest setting.

This case describes a pediatric patient successfully managed on venovenous ECMO after cardiac arrest from refractory anaphylaxis, highlighting the patient’s physiology and neurologic risk with venoarterial ECMO that contributed to the rapid change in cannulation strategy. This case adds to a small but growing body of literature suggesting that venovenous ECMO is possible in this context.

## Supplementary Material

## References

[1] Shaker MS: Anaphylaxis: Definition and criteria. J Food Allergy 2024; 6:26–31 39257603 10.2500/jfa.2024.6.240002 PMC11382771

[2] Cardona V Ansotegui IJ Ebisawa M: World allergy organization anaphylaxis guidance 2020. World Allergy Organ J 2020; 13:100472 33204386 10.1016/j.waojou.2020.100472 PMC7607509

[3] Peavy RD Metcalfe DD: Understanding the mechanisms of anaphylaxis. Curr Opin Allergy Clin Immunol 2008; 8:310–315 18596587 10.1097/ACI.0b013e3283036a90 PMC2683407

[4] Brown SGA: Clinical features and severity grading of anaphylaxis. J Allergy Clin Immunol 2004; 114:371–376 15316518 10.1016/j.jaci.2004.04.029

[5] Gupta RS Warren CM Smith BM: The public health impact of parent-reported childhood food allergies in the United States. Pediatrics 2018; 142:e20181235 30455345 10.1542/peds.2018-1235 PMC6317772

[6] Carlson R Schaeffer R Puri V: Hypovolemia and permeability pulmonary edeam associated with anaphylaxis. Crit Care Med 1981; 9:883–885 7318463 10.1097/00003246-198112000-00018

[7] Zhang Z Su X Liu C: Cardiac arrest with anaphylactic shock: A successful resuscitation using extracorporeal membrane oxygenation. Am J Emerg Med 2015; 33:130.e3–e4 10.1016/j.ajem.2014.06.034 25088440

[8] Weiss GM Fandrick AD Sidebotham D: Successful rescue of an adult with refractory anaphylactic shock and abdominal compartment syndrome with venoarterial extracorporeal membrane oxygenation and bedside laparotomy. Semin Cardiothorac Vasc Anesth 2015; 19:66–70 25552268 10.1177/1089253214564192

[9] Lee SY Chang CC Peng CM: Successful extracorporeal resuscitation after perioperative anaphylactic shock during living donor liver transplantation. Asian J Surg 2017; 40:317–319 25560544 10.1016/j.asjsur.2014.10.002

[10] Le HY Tien ND Son PN: Extracorporeal membrane oxygenation support in refractory anaphylactic shock after bee stings: A case report. Perfusion 2023; 38:1308–1310 35580365 10.1177/02676591221103540

[11] Ghanim D Adler Z Qarawani D: Takotsubo cardiomyopathy caused by epinephrine-treated bee sting anaphylaxis: A case report. J Med Case Rep 2015; 9:247 26518383 10.1186/s13256-015-0722-5 PMC4628238

[12] Sugiura A Nakayama T Takahara M: Combined use of ECMO and hemodialysis in the case of contrast-induced biphasic anaphylactic shock. Am J Emerg Med 2016; 34:1919.e1–e2 10.1016/j.ajem.2016.02.039 27021126

[13] Wang ML Chang CT Huang HH: Chlorhexidine-related refractory anaphylactic shock: A case successfully resuscitated with extracorporeal membrane oxygenation. J Clin Anesth 2016; 34:654–657 27687465 10.1016/j.jclinane.2016.07.002

[14] Wei J Zhang L Ruan X: Case report: Takotsubo syndrome induced by severe anaphylactic reaction during anesthesia induction and subsequent high-dose epinephrine resuscitation. Front Cardiovasc Med 2022; 9:842440 35369310 10.3389/fcvm.2022.842440 PMC8968145

[15] Grafeneder J Ettl F Warenits AM: Multi-phasic life-threatening anaphylaxis refractory to epinephrine managed by extracorporeal membrane oxygenation (ECMO): A case report. Front Allergy 2022; 3:934436 35966228 10.3389/falgy.2022.934436 PMC9372331

[16] Joseph J Bellezzo J: Refractory anaphylactic shock requiring emergent venoarterial extracorporeal membrane oxygenation in the emergency department: A case report. J Emerg Nurs 2022; 48:626–630 36109202 10.1016/j.jen.2022.08.002

[17] Ma DS Kim TH Keum MA: Management of cardiac arrest following anaphylactic reaction to cisatracurium using extracorporeal membrane oxygenation. Korean J Crit Care Med 2015; 30:42–45

[18] Chan-Dominy ACF Anders M Millar J: Extracorporeal membrane modality conversions. Perfusion 2015; 30:291–294 25070898 10.1177/0267659114544486

[19] Frederick AB Tresslar C Arenth J: Refractory anaphylactic shock to peanut ingestion in a pediatric patient requiring extracorporeal life support. Perfusion 2025:1–3 10.1177/02676591251360909 40631361

[20] Assouline B Combes A Schmidt M: Setting and monitoring of mechanical ventilation during venovenous ECMO. Crit Care 2023; 27:95 36941722 10.1186/s13054-023-04372-2 PMC10027594

[21] Shoskes A Migdady I Deshpande A: Comparison of neurologic complications of veno-arterial versus veno-venous extracorporeal membrane oxygenation: A systematic review and meta-analysis (1004). Neurology 2020; 94(15_Suppl):1004

[22] Rollins MD Hubbard A Zabrocki L: Extracorporeal membrane oxygenation cannulation trends for pediatric respiratory failure and central nervous system injury. J Pediatr Surg 2012; 47:68–75 22244395 10.1016/j.jpedsurg.2011.10.017

[23] Pavlushkov E Berman M Valchanov K: Cannulation techniques for extracorporeal life support. Ann Transl Med 2017; 5:70 28275615 10.21037/atm.2016.11.47 PMC5337209

[24] St-Arnaud C Thériault MM Mayette M: North-south syndrome in veno-arterial extra-corporeal membrane oxygenator: The other Harlequin syndrome. Can J Anaesth 2020; 67:262–263 31598907 10.1007/s12630-019-01501-w

[25] Shah NR Spencer BL Maselli KM: Lower extremity complications in children following femoral cannulation for extracorporeal membrane oxygenation. Perfusion 2024; 39:1692–1699 37977555 10.1177/02676591231216326

[26] Blakeslee-Carter J Shao C LaGrone R: Vascular complications based on mode of extracorporeal membrane oxygenation. J Vasc Surg 2022; 75:2037–2046.e2 35090988 10.1016/j.jvs.2022.01.078 PMC9133111

[27] Rath P Rath P Venkateshaiah L: 1521: Management of severe protamine-associated anaphylaxis with VV ECMO. Crit Care Med 2023; 52:S731–S731

[28] Scaravilli V Grasselli G Benini A: ECMO for intractable status asthmaticus following atracurium. J Artif Organs 2017; 20:178–181 27933398 10.1007/s10047-016-0940-7

[29] Garcia-Mendez JP Seelhammer TG Wieruszewski PM: VV-ECMO as a lifesaving measure for refractory bronchospasm in anaphylactic shock: A case report. Perfusion 2025:2676591251385891 41021782 10.1177/02676591251385891

[30] Maratta C Potera R van Leeuwen G: Extracorporeal life support organization (ELSO): 2020 Pediatric respiratory ELSO guideline. ASAIO J 2020; 66:975–979 32701626 10.1097/MAT.0000000000001223

[31] Bhardwaj A Miano T Geller B: Venovenous extracorporeal membrane oxygenation for patients with return of spontaneous circulation after cardiac arrest owing to acute respiratory distress syndrome. J Cardiothorac Vasc Anesth 2019; 33:2216–2220 31182376 10.1053/j.jvca.2019.04.020

[32] Guerguerian A Sano M Todd M: Pediatric extracorporeal cardiopulmonary resuscitation ELSO guidelines. ASAIO J 2021; 67:229–237 33627593 10.1097/MAT.0000000000001345

