# Supplementary figures and images for: Venovenous Extracorporeal Membrane Oxygenation After Cardiac Arrest Due to Refractory Anaphylaxis From Nut Ingestion
Source: Crit Care Explor. 2026 Apr 15;8(4):e1403. doi: 10.1097/CCE.0000000000001403 (PMC13086412; doi:10.1097/CCE.0000000000001403)

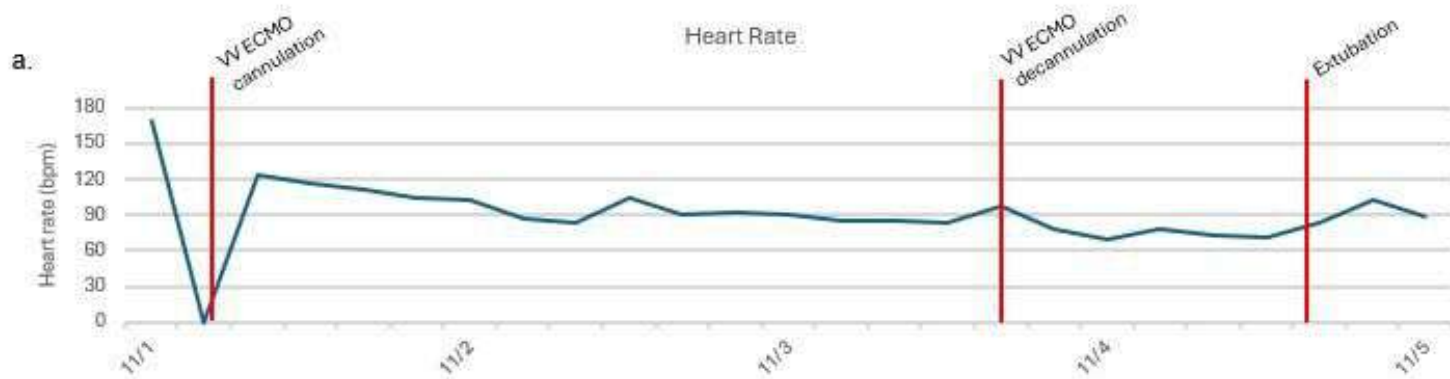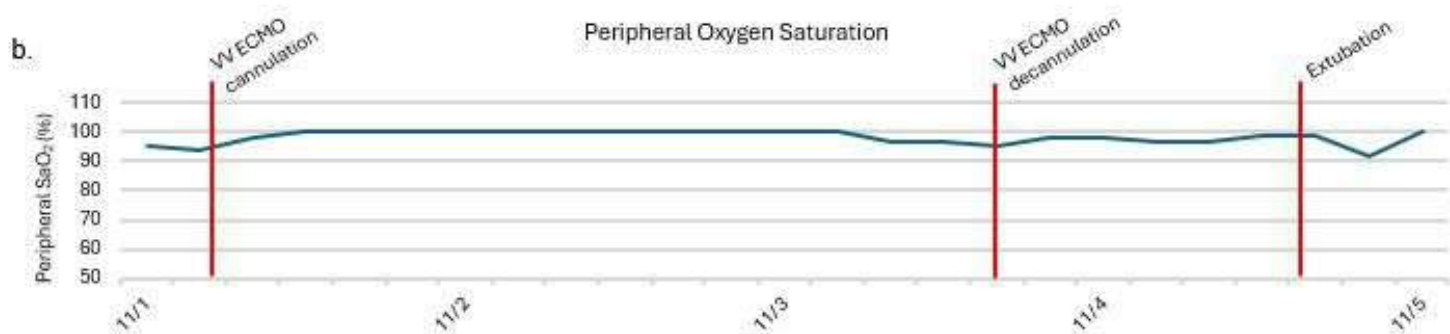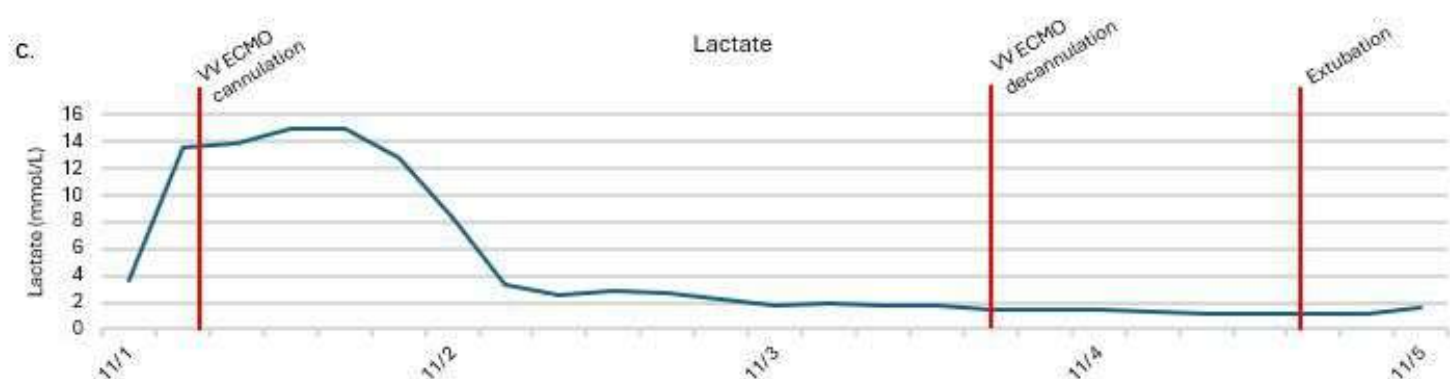

Supplement: Supplementary file 1 [file cc9-8-e1403-s001.pdf]
